# Effect of Hypochlorous Acid on Blepharitis through Ultrasonic Atomization: A Randomized Clinical Trial

**DOI:** 10.3390/jcm12031164

**Published:** 2023-02-01

**Authors:** Hong Zhang, Yuqing Wu, Xichen Wan, Yan Shen, Qihua Le, Pei Yang, Shuyun Zhou, Xujiao Zhou, Feng Zhou, Hao Gu, Jiaxu Hong

**Affiliations:** 1 Department of Ophthalmology, The Affiliated Hospital of Guizhou Medical University, Guiyang 550004, China; 2 Department of Ophthalmology, Eye and ENT Hospital, Fudan University, Shanghai 200437, China; 3 Institutes of Biomedical Sciences, Fudan University, Shanghai 200032, China

**Keywords:** hypochlorous acid (HOCL), blepharitis, meibomian gland dysfunction, dry eye, ultrasonic atomization

## Abstract

Purpose: To evaluate the efficacy and safety of eyelid hygiene using topical 0.01% hypochlorous acid (HOCL) through ultrasonic atomization after 2 weeks in patients with blepharitis. Design: Randomized controlled trial. Methods: Patients with blepharitis were randomized into two groups: topical 0.01% HOCL through ultrasonic atomization (HOCL group, 42 eyes) or eyelid scrubs (control group, 37 eyes). Patients in both groups received warm compresses twice daily and topical 0.5% levofloxacin three times a day. Primary outcomes were the ocular surface disease index scores (OSDI), lid margin redness, lid margin abnormalities, meibum expressibility, meibum quality, and noninvasive breakup time after 2 weeks. Secondary outcomes were conjunctiva redness, corneal fluorescein staining, and tear meniscus height. A questionnaire of treatment adherence with a free response section was administered to confirm patient compliance and comments. Results: Sixty-seven participants participated in this study. Both groups show an improvement in all primary outcomes, while statistically significant improvements in OSDI, lid margin redness, lid margin abnormality, meibum expressibility and quality are only limited to the HOCL group after 2 weeks of treatment (*p* < 0.05, *p* < 0.05, *p* < 0.001, *p* < 0.001 and *p* < 0.001, respectively). Subgroup analysis in HOCL reveals that only the change in lid margin abnormality and meibum expressibility in the mild–moderate meibomian glands loss patients at baseline has a statistically significant difference *p* < 0.05). Multiple linear regression shows that the improvement in OSDI is negatively associated with meibum expressibility score at the baseline (95% CI [−28.846, −1.815], *p* = 0.028). The patient compliance is 7.1 ± 2.0 in the HOCL group and 7.1 ± 1.8 in the control group (*p* > 0.05). No adverse events are reported. Conclusion: Topical 0.01% HOCL through ultrasonic atomization is a tolerable and effective eyelid hygiene treatment for blepharitis.

## 1. Introduction

Blepharitis is an ophthalmic condition that is commonly encountered in clinical practice [1], with a reported prevalence of 37% and 47% in ophthalmology and optometry clinics in the United States, respectively [2]. It is characterized by the over-colonization of bacteria [3] and chronic inflammation involving the eyelashes, periocular skin, eyelid margin, and meibomian glands, which has been shown to disrupt tear film stability and frequently lead to the development of meibomian gland dysfunction (MGD)-related dry eye disease (DED) and other ocular surface inflammatory diseases [4,5]. In the most severe cases, blepharitis could cause vision-threatening conditions [6]. Current treatment of blepharitis often includes using a warm compress, eyelid cleansing, and topical antibiotic and anti-inflammatory therapy [1]. Among them, eyelid cleansers are recommended as a long-term management for symptomatic relief [7,8]. However, cleansers containing tea tree oil may induce ocular discomfort and discourage patients from continuous use [9,10]. Concerns and inconvenience around diluted baby shampoo also impacted patient adherence, including the potential presence of pro-inflammation agents in formulation [11], the risk of contamination during dilution, and the damage of diluted solution during storage [12]. These issues highlighted the urgent need for a novel eyelid hygiene product with efficacy and safety.

Hypochlorous acid (HOCL), a natural compound released by phagocytes [13], is well-known for its wide antimicrobial spectrum [14]. The use of 0.01% HOCL has been identified as being as effective as 5% povidone iodine in reducing bacterial load on the lid margins [15]. One study examined 0.01% HOCL in patients with anterior blepharitis and the results showed that 0.01% HOCL decreased over 90% of the bacterial load without altering the diversity of bacterial species [3]. Several previous studies identified the ocular safety profile of 0.01% HOCL [16,17,18], and one reported that 0.01% was of the highest degree of patients’ comfort among several eyelid cleansers [18].

Ultrasonic atomization (UA) is a procedure that damages liquid surface tension and atomizes drops into discrete elements through ultrasonic vibration [19], enabling droplets to permeate the ocular surface and enhance drug delivery [20]. Topical uses of 0.01% HOCL [20] and *houttuynia* [21] through UA have previously been demonstrated to ameliorate MGD-related DED. However, whether the use of 0.01% HOCL through ultrasonic atomization can alleviate blepharitis has not been studied.

In this prospective, multi-center, assessor-masked, placebo-controlled, randomized clinical trial, we evaluated the effect of 0.01% HOCL through UA on the improvement in OSDI scores, lid margin redness, lid margin abnormality, meibum expressibility, meibum quality, and noninvasive breakup time (NIBUT) in patients with blepharitis after 2 weeks of treatment. We then compared the result with a control group that was treated with eyelid scrubs.

## 2. Materials and Methods

### 2.1. Subjects

This prospective, 2 week, assessor-masked, randomized trial conformed to the tenets of the Declaration of Helsinki and was approved by the Institutional Review Board of the Eye and ENT Hospital of Fudan University (EENTIRB-20190301). The trial was registered with Clinical-Trials.gov (identification number NCT05608980).

Inclusion criteria included patients who were 18 years or older with clinical signs of blepharitis in a slit lamp examination (such as eyelash crusting, collarettes; eyelid margin thickening, rounding, notching, and telangiectasia; and/or meibomian gland capping), and no contact lens wear or use of topical/systemic medications known to affect the eye for at least 2 weeks prior to the baseline assessment or during the treatment period. Exclusion criteria included any history of eyelid surgery, a history of ophthalmic surgery in the previous 3 months or during the treatment period, history of intolerance or hypersensitivity to the study medications, pregnancy, and lactating women.

**Figure 1:**
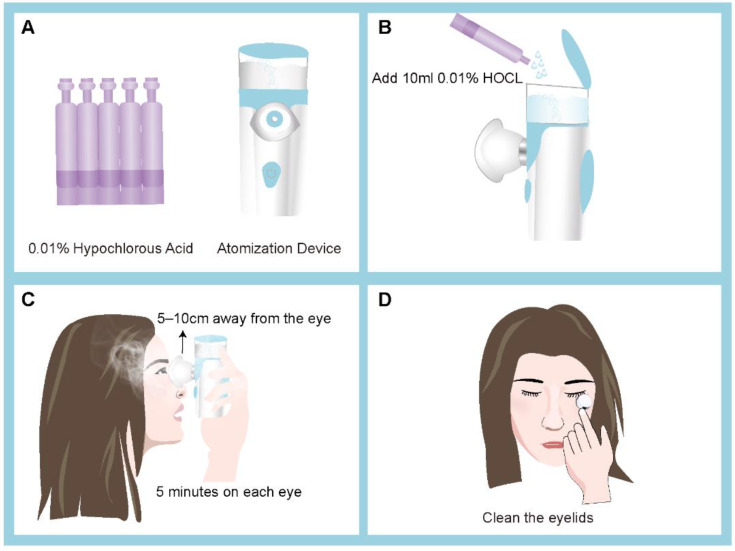
The portable atomization device and how it is used on a patient. Illustration of using 0.01% hypochlorous acid (HOCL) via an atomization device (**A,B**), which can be easily sterilized with alcohol wipes. Patients with blepharitis using 0.01% HOCL through ultrasonic atomization for treatment (**C,D**).

A total of 79 eligible participants were recruited from the Eye and ENT Hospital of Fudan University, the Shuguang Hospital, and the New Insight Hospital between 1 July 2022 and 31 September 2022, which exceeded the sample size requirements for the desired study power The sample size was calculated using continuous data, with ocular surface disease index (OSDI) scores as an endpoint, through an independent sample study sample size calculator with NCSS PASS 2002 (Kaysville, UT, USA). According to a previous study [2], a side effect of 0.8 was determined, at 80% power (β = 0.2) and with a two-sided statistical significance level of 5% (α = 0.05). A minimum of 25 participants was required in each group. Considering a rate of 30%, at least 36 patients should be included in each group. The patients were randomly allocated into two groups (Figure 1). The right eye was enrolled as the test eye. In the HOCL group, 42 eyes of 42 patients topically received eyelid hygiene with 0.01% HOCL (O’FORLA, Shanghong, Shanghai, China) through a portable ultrasonic atomizer (Nano eye sprayer, Yijiekang, Shenzhen, China) once daily for 2 weeks. Patients included in this study were trained at clinic for their first time about how to use the portable ultrasonic atomizer or eye scrubs with the help of care providers (P.Y. and S.Z.) and given written instructions, after which they could take the device or eye scrubs home and performed the treatment by themselves during the following periods. In HOCL group, patients were instructed to apply 10 mL of 0.01% HOCL into the portable ultrasonic atomizer, hold it 5–10 cm in front of the eye, keeping each eye open for 5 min (Figure 1). In the control group, 37 eyes of 37 patients received eyelid scrubs twice daily for 2 weeks. Patients were provided with cotton swabs and 0.9% saline. After applying the saline into cotton swabs, patients were instructed to gently rub along the length of the upper and lower eyelids for 30 s with the cotton swabs.

As eyelid hygiene alone is insufficient to modulate the inflammatory process in blepharitis, patients in both groups underwent warm compresses twice daily and topical 0.5% levofloxacin (Cravit, Santen, Osaka, Japan) three times a day. Seven patients (seven eyes) in the HOCL group and five patients (five eyes) in the control group were lost to follow-up. Measurements of the remaining 67 eyes of 67 patients were used for statistical analysis. 

### 2.2. Outcome Measures 

The primary end points after 2 weeks of treatment included the following: a mean reduction in clinical symptoms measured by OSDI scores [22], changes in the score of lid margin redness and lid margin abnormalities, and meibum expressibility, meibum quality, and a change in NIBUT. Secondary end points after 2 weeks of treatment included a change in the mean reduction in conjunctiva redness, a mean reduction in corneal epitheliopathy as measured by corneal fluorescein staining (CFS), and changes in tear meniscus height (TMH).

Clinical assessments were conducted at baseline and day 14 of the treatment period. The assessors conducting clinical measurements and analyzed the results were masked to the treatment randomization. Clinical measurements were performed in ascending order of invasiveness in order to minimize the impact on tear film physiology for subsequent tests [23]. Safety endpoints included ocular and systemic adverse events related to the 0.01% HOCL. A questionnaire of medication adherence was administered to patients at day 14 [24], with a total score ranging from 0–8 and poor adherence for a score <6, fair adherence for a score of 6–7, and good adherence for a score of 8. There was also a free response section where patients could input any comment.

Lid margin redness was evaluated by slit lamp examinations, using a 4 point rating scale, with 0 being absent and 3 being the most severe [25].

The lid margin abnormality score was calculated under a slit lamp to assess the meibomian glands (MGs) morphologic lid features, based on the sum of the following four signs [26,27]: lid margin irregularities, telangiectasia, orifice plugging, and displacement of mucocutaneous junction, with each sign receiving a score of 1. The total score ranged from 0 to 4.

Meibum expressibility and quality were measured by using firm digital pressure applied over five lower lid glands. Expressibility was scored as the following: grade 0 if all five glands were expressible; grade 1 if three to four glands were expressible; grade 2 if one or two glands were expressible; and grade 3 if zero glands were expressible [28]. Meibum quality was graded as grade 0 if clear; grade 1 if cloudy; grade 2 if cloudy with granular debris; and grade 3 if a thick-like toothpaste [27,29].

Noninvasive tear film breakup time, meibography, conjunctiva redness, and TMH were assessed using the Keratograph 5M (Oculus, Wetzlar, Germany). The NIBUT included the first NIBUT (NIBUT-1st) and the average NIBUT (NIBUT-avg). The NIBUT-1st was measured using automated detection of the first breakup while the participant maintained fixation and was requested to refrain from blinking [23]. When observing MGs, the upper and lower eyelids were everted and graded. The meiboscore were graded as: 0, no loss of MGs; 1, less than one-third loss of glands; 2, one-third to two-thirds loss of glands; and 3, more than two-thirds loss of glands. The total meiboscore of the upper and lower eyelids ranged from 0–6 [30,31,32]. Conjunctiva redness was assessed by automated objective evaluation of the high magnification digital imaging according to the proprietary JENVIS grading scale from 0 to 4 [12]. The TMH was recorded as an average of three measurements near the center of the lower meniscus [33].

Cornea fluorescein staining was examined using a slit lamp with a maximum cobalt blue light. Cornea was divided into five sectors (central, superior, inferior, nasal, and temporal), each scored 0–3, with a total score ranging from 0 to15 [23].

### 2.3. Statistical Analysis

Statistical analysis was conducted using IBM SPSS version 21 (New York, NY, USA). The main variables were ordinal data. The distribution of continuous variables was examined by K–S test. The between-group analysis was assessed using the chi-square text, *t*-test, or the Wilcoxon signed-rank test. Variables at baseline and 2 weeks were tested with a paired Wilcoxon signed-rank test. Multiple linear regression was conducted to verify the predictors of the change in OSDI. Differences were considered statistically significant when the *p*-values were less than 0.05.

## 3. Results

**Figure 2:**
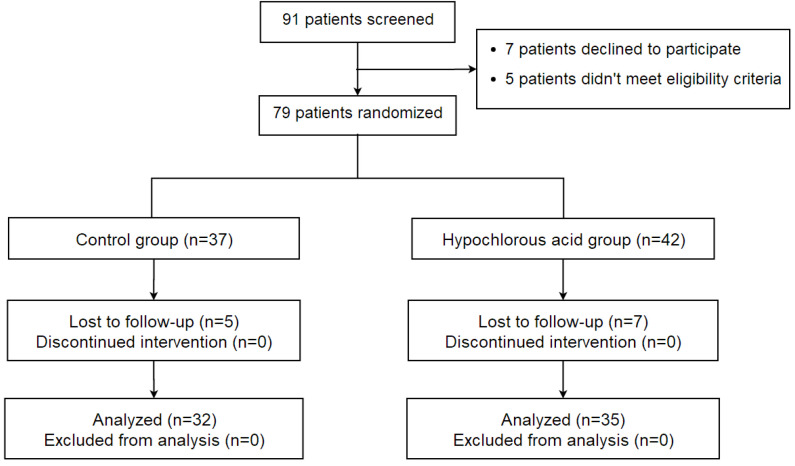
Flow diagram according to the consolidated standards of reporting trials (CONSORT) statement, showing enrollment, randomization, and patient flow in this study of 0.01% hypochlorous acid for blepharitis.

Sixty-seven participants completed the study. The flow of patients through the study is shown in Figure 2. There were 35 participants in the HOCL group (12 males, 34.3%; mean age, 41.3 ± 15.9 years) and 32 participants (13 males, 40.6%; mean age, 41.1 ± 13.6 years) in the control group. Two groups were comparable at baseline at demographic characteristics and ocular surface parameters (Table 1), except for the poorer meibum expressibility in the HOCL group compared to the control group (2.3 ± 0.6 vs. 1.8 ± 0.7, *p* < 0.05).

**Figure 3:**
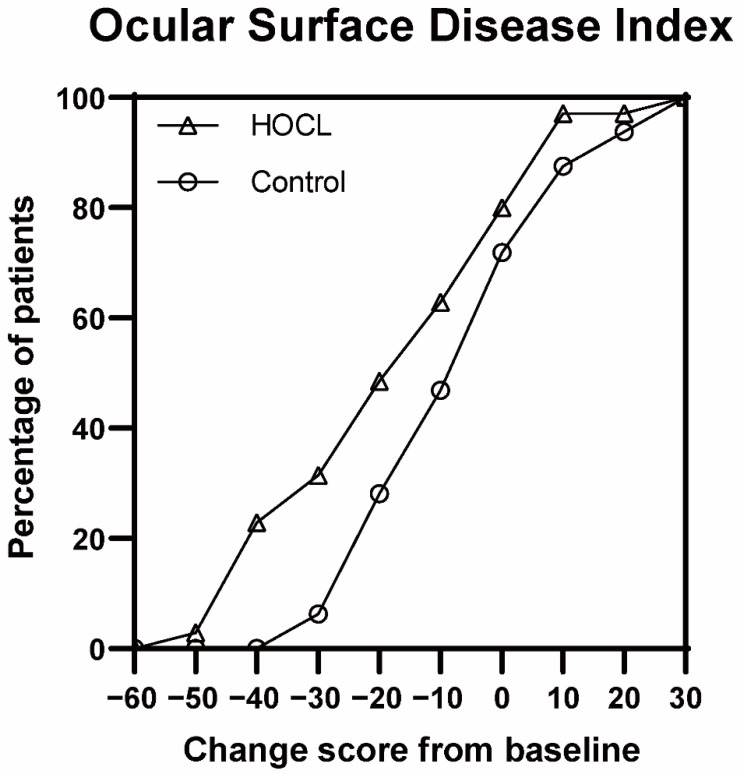
Cumulative distributions of the primary endpoint in the study of 0.01% hypochlorous acid (HOCL) for blepharitis. Shown is the cumulative distribution of the change score from the baseline of the ocular surface disease index (OSDI). Proportion of patients in the HOCL group show more changes than the control group.

**Figure 4:**
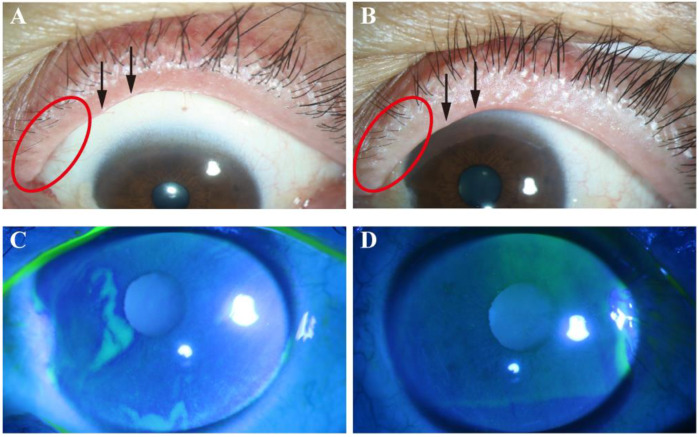
Representative images of lid margin findings and corneal fluorescein staining in hypochlorous acid group. Note that obvious lid margin redness and plugging of gland orifices are observed at baseline (**A**) and alleviation of these signs occurs after 2 weeks (**B**). There was a confluent spot staining at baseline (**C**), while no punctate staining was observed after 2 weeks of treatment (**D**). Red circles indicate lid margin redness; arrows indicate plugging.

**Figure 5:**
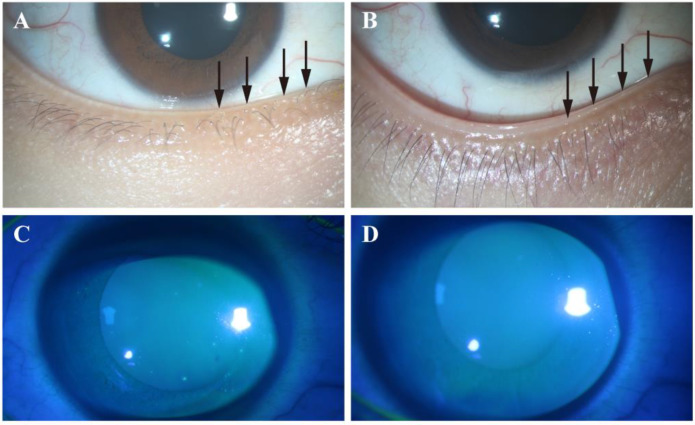
Representative images of lid margin findings and corneal fluorescein staining in the control group. Note that plugging of gland orifices is observed at baseline (**A**), and there were no obvious changes in plugging after 2 weeks (**B**). There was confluent spot staining at baseline (**C**), which was still observed after 2 weeks of treatment (**D**). Arrows indicate plugging.

The results of improvements in the ocular surface parameters after 2 weeks of treatment are shown in Table 2. The 0.01% HOCL treatment results in an improvement in OSDI from baseline to 2 weeks. The improvement in OSDI changes from a score of 35.6 ± 19.3 to 15.3 ± 9.7 (*p* < 0.001; Supplementary Table S1) in the HOCL group, which shows a statistical difference over the control group (−20.3 ± 20 vs. −9.6 ± 15.3, *p* < 0.05; Table 2). To better understand the beneficial effect of treatment in the HOCL group, the cumulative distribution of the change score from baseline to 2 weeks is provided in Figure 3 [34]. This figure reveals that 48.6% in the HOCL group achieve a changed OSDI score from baseline that is less than −20, while only 28.1% of the eyes in the control group meet this condition. Patients in the HOCL group also show a significant improvement in lid margin redness after 2 weeks of treatment when compared to the control group (−1.0 ± 0.7 vs. −0.6 ± 0.5, *p* = 0.011, Table 2; Figure 4 and Figure 5), as well as in lid margin abnormality, meibum expressibility, and meibum quality (Table 2). In terms of secondary outcomes, a significant reduction in conjunctiva redness and CFS is also observed in the HOCL group in comparison to the control group (*p* < 0.001 and *p* < 0.05; Table 2). There is no significant difference in improvements between the HOCL group and control group in the change of NIBUT-1st, NIBUT-avg, and TMH (*p* > 0.05). Two groups are comparable at compliance (*p* > 0.05; Table 2). No adverse events were reported during the study.

To further understand the efficacy of HOCL, a subgroup analysis was performed in the HOCL group according to the degree of baseline meibomian gland loss. Subjects were divided into severe meibomian gland loss (MGL) (total meiboscore at baseline > 4) group and mild-to-moderate MGL (total meiboscore at baseline ≤ 4) group. In both groups, lid margin redness and lid margin abnormality, as well as meibum expressibility and quality show significant improvements after 2 weeks of treatment (*p* < 0.05; Supplementary Table S2). However, only the improvement in OSDI and NIBUT is limited to patients with mild-to-moderate MGL. Analysis between the two groups demonstrates that only the change in lid margin abnormality and meibum expressibility in the mild–moderate MGL patients at baseline in HOCL group have a statistically significant difference (*p* < 0.05; Table 3).

A multiple linear regression model, using the change in OSDI as the dependent variable, was performed to adjust for an intra-patient correlation of the baseline ocular surface parameters in the HOCL group [35]. Analysis reveals that changes in OSDI are negatively associated with the meibum expressibility score (95% CI [−28.846, −1.815], *p* = 0.028) in the HOCL group (Table 4). All other baseline parameters, including age, sex, lid margin redness, lid margin abnormality, or meibum quality do not independently predict the OSDI outcome.

## 4. Discussion

The current study demonstrates that, compared with traditional eyelid scrubs, eyelid hygiene with 0.01% HOCL through ultrasonic atomization significantly improves the OSDI score, which is consistent with improving the lid margin redness and lid margin abnormality, as well as meibum expressibility and quality. In addition, no drug-related discomfort and adverse side events were reported.

The main point of care for a patient with blepharitis should be symptom relief [36], and OSDI is commonly used as the primary outcome for evaluating treatment efficacy [12,33]. Our results show that there is a statistically significant improvement in OSDI scores in the HOCL group, with a reduction of 20.3 ± 20 scores, while there is only a reduction of 9.6 ± 15.3 score in the control group. These results are in agreements with previous studies, whereby topical 0.01% HOCL spray after intravitreal injection effectively improves patients’ quality of life [37]. The effect of 0.01% HOCL has also been identified in dry eye patients, which results in a relief of symptoms over a 30 day period of use [38]. Although the pathogenesis of blepharitis remains to be completely understood yet, it is well-known that the bacterial over-colonization is associated with activating the inflammatory responses and hypersensitivity [7,8,33]. HOCL acts on anti-bacterial activity through protein and lipid peroxidation, and exhibits its anti-inflammatory activity by neutralizing inflammatory mediators in the body [3,15,20]. This is consistent with our findings that, after 2 weeks of the 0.01% HOCL treatment, there is a satisfactory reduction in comparison with control groups, with a reduction in lid margin redness (1.0 ± 0.7 vs. 0.6 ± 0.5), lid margin abnormality (0.7 ± 0.6 vs. 0.2 ± 0.4), meibum quality (1.3 ± 0.6 vs. 0.7 ± 0.6), and expressibility (1.1 ± 0.7 vs. 0.5 ± 0.6), which are all vital indicators of eyelid inflammation [39]. Similarly, 0.01% HOCL has also been reported to decrease the expression of MMP-9 and IL-2 in the tear of MGD-related DED patients [20]. In addition, patients in the HOCL group show a better improvement in CFS score than the control group (−0.5 ± 1.0 vs. −0.1 ± 0.3, *p* < 0.05), which may be explained by HOCL being effective in controlling the biofilms, stabilizing the tear film, and promoting healing of the ocular epithelium [40]. Interestingly, in the HOCL group, there are significant improvements in lid margin abnormality and meibum expressibility in patients with mild-to-moderate MGL when compared to patients with severe MGL. On the other hand, the multiple linear regression model suggests that meibum expressibility at baseline in the HOCL group is associated with OSDI score reduction. These phenomena indicate that patients with mild-to-moderate MGL could be the potential target population of topical 0.01% HOCL treatment and benefit most from this adjuvant therapy. In agreement with our results, a recent study found that 0.01% HOCL increased the tear breakup time of MGD-DED [20]. Together, based on previous evidence [39], we propose that topical 0.01% HOCL might help to stabilize the ocular surface by improving lid morphology, and meibum quality and quantity, improvements in which subsequently lead to a symptomatic relief.

The ultrasonic atomization device shows great advantages in enhancing drug utilization by creating a ventilated, high-humidity, and transparent mirror room around the ocular surface [20]. Through the ultrasonic atomization process, liquid sheets disintegrate into fine droplets, enabling droplets to permeate and evenly distribute in the ocular surface [19], which contributes to drug delivery. On the other hand, the portable ultrasonic atomizer in our study is much more convenient than traditional ultrasonic atomizer, which enables patients to perform treatment at home or the office, and not bother with a visit to the hospital. Our study confirms that a topical application of 0.01% HOCL via ultrasonic atomization is well-tolerated. Patient compliance to lid hygiene has been an issue in managing blepharitis, impacting therapeutic inefficacy and inconvenience [41]. The treatment compliance in the current study of both groups is satisfactory, as confirmed by the questionnaire. In the patients’ free comments section, a 35 year old female in the HOCL group stated “I appreciated the progress of atomization. It was quite enjoyable and relaxed, like having a facial mask”. The preference for a portable ultrasonic atomizer may encourage patients to practice regular eyelid hygiene in the long term.

One limitation in our study is the short-term treatment period. It is possible that 2 weeks of treatment with once daily application was insufficient to develop cumulative effects. Future studies with a larger number of patients and longer treatment periods are warranted in order to determine the optimal frequency and period of HOCL treatment. In addition,, the portable ultrasonic atomizer lacked a heated element and failed to achieve temperature control of liquid. A warm temperature of atomization could help to promote meibum expressibility.

In summary, 0.01% HOCL through ultrasonic atomization for 5 min on each eye was effective and safe in treating blepharitis after 2 weeks, which could serve as a potential adjuvant therapy for blepharitis, in addition to the basic treatment with topical 0.5% levofloxacin plus warm compresses.

## Figures and Tables

**Table 1:** Demographic characteristics and ocular surface parameters of hypochlorous acid and control groups at baseline.

Parameters	Mean ± SD		*p* Value
	HOCL	Control	
Participants	35	32	
Male, n (%) ^a^	12 (34.3)	13 (40.6)	0.592
Age, year ^b^	41.3 ± 15.9	41.1 ± 13.6	0.959
Primary outcomes			
OSDI ^b^	35.6 ± 19.3	31.8 ± 15.3	0.375
Lid margin redness ^c^	1.5 ± 0.6	1.7 ± 0.5	0.053
Lid margin abnormality ^c^	1.3 ± 0.5	1.3 ± 0.4	0.894
Meibum expressibility ^c^	2.3 ± 0.6	1.8 ± 0.7	**0.004**
Meibum quality ^c^	2.0 ± 0.3	1.9 ± 0.4	0.504
NIBUT-1st, s ^c^	9.2 ± 4.9	7.6 ± 4.9	0.160
NIBUT-avg, s ^c^	11.6 ± 4.3	10.7 ± 4.2	0.309
Secondary outcome			
Conjunctiva redness ^c^	1.3 ± 0.6	1.4 ± 0.4	0.388
CFS score ^c^	0.5 ± 1.0	0.2 ± 0.5	0.490
TMH, mm ^c^	0.3 ± 0.1	0.2 ± 0.1	0.744
Meibography score ^c^			
Upper lid	1.6 ± 0.9	1.8 ± 0.7	0.365
Lower lid	1.6 ± 0.8	1.4 ± 0.7	0.353
Total	3.2 ± 1.5	3.2 ± 1.2	0.979

Abbreviations: HOCL, hypochlorous acid; SD, standard deviation; OSDI, ocular surface disease index; NIBUT-1st, the first time of noninvasive breakup time; NIBUT-avg, the mean time of noninvasive breakup time; CFS, corneal fluorescein staining; TMH, tear meniscus height. ^a^ *p* values based on χ^2^ and ^b^ *p* values based on *t* test. ^c^ *p* values based on Wilcoxon rank sum tests. *p* values marked in **bold** indicate significant.

**Table 2:** Comparison of improvements in ocular surface parameters after 2 weeks treatment and patient compliance between hypochlorous acid group and control group.

Parameters	HOCL	Control	*p* Value
Primary outcomes			
OSDI ^a^	−20.3 ± 20	−9.6 ± 15.3	**0.018**
Lid margin redness ^b^	−1.0 ± 0.7	−0.6 ± 0.5	**0.011**
Lid margin abnormality ^b^	−0.7 ± 0.6	−0.2 ± 0.4	**<0.001**
Meibum expressibility ^b^	−1.1 ± 0.7	−0.5 ± 0.6	**<0.001**
Meibum quality ^b^	−1.3 ± 0.6	−0.7 ± 0.6	**<0.001**
NIBUT-1st, s ^b^	2.5 ± 5.2	1.4 ± 4.0	0.422
NIBUT-avg, s ^b^	1.7 ± 4.1	0.6 ± 2.7	0.608
Secondary outcomes			
Conjunctiva redness ^b^	−0.2 ± 0.4	0.1 ± 0.4	**<0.001**
CFS score ^b^	−0.5 ± 1	−0.1 ± 0.3	**0.047**
TMH, mm ^a^	0.0 ± 0.1	0.0 ± 0.1	0.942
Compliance ^b^	7.1 ± 2.0	7.1 ± 1.8	0.887

Abbreviations: HOCL, hypochlorous acid; OSDI, ocular surface disease index; NIBUT-1st, the first time of noninvasive breakup time; NIBUT-avg, the mean time of noninvasive breakup time; CFS, corneal fluorescein staining; TMH, tear meniscus height. ^a^ *p* values based on *t* test. ^b^ *p* values based on Wilcoxon rank sum tests. *p* values marked in **bold** indicate significant.

**Table 3:** Comparison of improvements in ocular surface parameters after 2 weeks treatment according to the baseline meibomian gland loss in the hypochlorous acid (HOCL) group.

Parameters	Baseline Mild–Moderate MGL n = 29	Baseline Severe MGL n = 6	*p* Value
Primary outcomes			
OSDI ^a^	−20.7 ± 20.6	−18.2 ± 18.3	0.780
Lid margin redness ^b^	−0.9 ± 0.7	−1.3 ± 0.5	0.141
Lid margin abnormality ^b^	−0.7 ± 0.6	−1.2 ± 0.4	**0.044**
Meibum expressibility ^b^	−1.0 ± 0.7	−1.7 ± 0.5	**0.013**
Meibum quality ^b^	−1.3 ± 0.7	−1.5 ± 0.6	0.364
NIBUT-1st, s ^a^	2.8 ± 5.2	0.8 ± 5.2	0.393
NIBUT-avg, s ^a^	1.6 ± 4.1	2.1 ± 4.4	0.808
Secondary outcomes			
Conjunctiva redness ^a^	−0.2 ± 0.3	−0.6 ± 0.7	0.214
CFS score ^b^	−0.5 ± 1.0	−0.7 ± 1.2	0.531
TMH, mm ^a^	0.0 ± 0.1.	0.1 ± 0.0	**0.003**
Compliance ^b^	6.9 ± 2.2	7.8 ± 0.6	0.442

Abbreviations: MGL, meibomian gland loss; OSDI, ocular surface disease index; NIBUT-1st, the first time of noninvasive breakup time; NIBUT-avg, the mean time of noninvasive breakup time; CFS, corneal fluorescein staining; TMH, tear meniscus height. ^a^ *p* values based on paired-*t* test. ^b^ *p* values based on paired Wilcoxon rank sum tests. *p* values marked in **bold** indicate significant.

**Table 4:** Results of a multiple linear regression analysis examining the impact of relative variables at baseline on the changes in ocular surface disease index.

Predictor	Unstandardized Coefficients Beta	Standardized Coefficients Beta	95%CI		*p* Value
			Lower	Upper	
Age	−0.319	−0.253	−0.739	0.101	0.131
Sex	−0.050	−0.001	−14.406	14.307	0.994
Lid margin redness	−7.350	−0.206	−23.281	8.581	0.353
Lid margin abnormality	12.563	0.326	−5.994	31.120	0.176
Meibum expressibility	−15.331	−0.477	−28.846	−1.815	**0.028**
Meibum quality	1.017	0.017	−20.981	23.015	0.925

*p* values marked in **bold** indicate significant.

## Data Availability

The authors confirm that the data supporting the findings are available from the corresponding author upon reasonable request.

## Supplementary Materials

The following supporting information can be downloaded at: https://www.mdpi.com/article/10.3390/jcm12031164/s1, Table S1. Changes of clinical outcomes from baseline to 2 weeks after treatment in hypochlorous acid (HOCI) group and control group. Table S2. Changes of clinical outcomes from baseline to 2 weeks after treatment according to baseline meibomian gland loss in the hypochlorous acid (HOCI) group.

## References

[1] Amescua G., Akpek E.K., Farid M., Garcia-Ferrer F.J., Lin A., Rhee M.K., Varu D.M., Musch D.C., Dunn S.P., Mah F.S. (2018). Blepharitis Preferred Practice Pattern^®^. Ophthalmology.

[2] Lemp M.A., Nichols K.K. (2009). Blepharitis in the United States 2009: A Survey-based Perspective on Prevalence and Treatment. Ocul. Surf..

[3] Stroman D., Mintun K., Epstein A., Brimer C., Patel C., Branch J., Najafi-Tagol K. (2017). Reduction in bacterial load using hypochlorous acid hygiene solution on ocular skin. Clin. Ophthalmol..

[4] Bron A.J., de Paiva C.S., Chauhan S.K., Bonini S., Gabison E.E., Jain S., Knop E., Markoulli M., Ogawa Y., Perez V. (2017). TFOS DEWS II pathophysiology report. Ocul. Surf..

[5] Schaumberg D.A., Nichols J.J., Papas E.B., Tong L., Uchino M., Nichols K.K. (2011). The International Workshop on Meibomian Gland Dysfunction: Report of the Subcommittee on the Epidemiology of, and Associated Risk Factors for, MGD. Investig. Opthalmol. Vis. Sci..

[6] O’Gallagher M., Bunce C., Hingorani M., Larkin F., Tuft S., Dahlmann-Noor A. (2017). Topical treatments for blepharokeratocon-junctivitis in children. Cochrane Database Syst. Rev..

[7] Pflugfelder S.C., Karpecki P.M., Perez V.L. (2014). Treatment of Blepharitis: Recent Clinical Trials. Ocul. Surf..

[8] Duncan K., Jeng B.H. (2015). Medical management of blepharitis. Curr. Opin. Ophthalmol..

[9] Gao Y.-Y., A Di Pascuale M., Elizondo A., Tseng S.C.G. (2007). Clinical Treatment of Ocular Demodecosis by Lid Scrub with Tea Tree Oil. Cornea.

[10] Navel V., Mulliez A., Benoist d’Azy C., Baker J.S., Malecaze J., Chiambaretta F., Dutheil F. (2019). Efficacy of treatments for Demodex blepharitis: A systematic review and me-ta-analysis. Ocul. Surf..

[11] Polack F.M., Goodman D.F. (1988). Experience With a New Detergent Lid Scrub in the Management of Chronic Blepharitis. Arch. Ophthalmol..

[12] Sung J., Wang M.T.M., Lee S.H., Cheung I.M., Ismail S., Sherwin T., Craig J.P. (2018). Randomized double-masked trial of eyelid cleansing treatments for blepharitis. Ocul. Surf..

[13] Hurst J.K. (2012). What really happens in the neutrophil phagosome?. Free Radic. Biol. Med..

[14] NovaBay Pharmaceuticals, Inc. https://novabay.com/wp-content/uploads/2015/05/avenova-with-neutrox-first-and-only-product-in-ophthalmology-containing-pure-hypochlorous-acid-compared-with-otc-product.pdf.

[15] Gonzalez L.A., Kowalski R.P., Dhaliwal D.K. (2018). Efficacy of topical 0.01% hypochlorous acid in reducing bacterial flora on lid margins compared to topical 5% povidone-iodine. Investig. Ophthalmol. Vis. Sci..

[16] Romano G.L., Lazzara F., Conti F., Giunta S., Drago F., Bucolo C. (2021). Ocular safety profile of a new formulation based on hypo-chlorous acid in rabbit eye. Investig. Ophthalmol. Vis. Sci..

[17] Gold M.H., Andriessen A., Bhatia A.C., Bitter P., Chilukuri S., Cohen J.L., Robb C.W. (2020). Topical stabilized hypochlorous acid: The future gold standard for wound care and scar management in dermatologic and plastic surgery procedures. J. Cosmet. Dermatol..

[18] Ngo W., Jones L., Bitton E. (2018). Short-Term Comfort Responses Associated with the Use of Eyelid Cleansing Products to Manage Demodex folliculorum. Eye Contact Lens Sci. Clin. Pract..

[19] Avvaru B., Patil M.N., Gogate P.R., Pandit A.B. (2006). Ultrasonic atomization: Effect of liquid phase properties. Ultrasonics.

[20] Li Z., Wang H., Liang M., Li Z., Li Y., Zhou X., Kuang G. (2022). Hypochlorous Acid Can Be the Novel Option for the Meibomian Gland Dysfunction Dry Eye through Ultrasonic Atomization. Dis. Markers.

[21] Liu Z., Jin M., Li Y., Liu J., Xiao X., Bi H., Liu Z. (2020). Efficacy and safety of houttuynia eye drops atomization treatment for meibomian gland dysfunc-tion-related dry eye disease: A randomized, double-blinded, placebo-controlled clinical trial. J. Clin Med..

[22] Stapleton F., Alves M., Bunya V.Y., Jalbert I., Lekhanont K., Malet F., Na K.-S., Schaumberg D., Uchino M., Vehof J. (2017). TFOS DEWS II epidemiology report. Ocul. Surf..

[23] Wolffsohn J.S., Arita R., Chalmers R., Djalilian A., Dogru M., Dumbleton K., Gupta P.K., Karpecki P., Lazreg S., Pult H. (2017). TFOS DEWS II Diagnostic Methodology report. Ocul. Surf..

[24] Moon S.J., Lee W.-Y., Hwang J.S., Hong Y.P., Morisky D.E. (2017). Accuracy of a screening tool for medication adherence: A systematic review and meta-analysis of the Morisky Medication Adherence Scale-8. PLoS ONE.

[25] Li A.L.-W., Li S.L., Kam K.W., Young A.L. (2022). Randomised assessor-masked trial evaluating topical manuka honey (Optimel) in treatment of meibomian gland dysfunction. Br. J. Ophthalmol..

[26] Arita R., Itoh K., Maeda S., Maeda K., Furuta A., Fukuoka S., Tomidokoro A., Amano S. (2009). Proposed Diagnostic Criteria for Obstructive Meibomian Gland Dysfunction. Ophthalmology.

[27] Wu K.-I., Chen C.-Y., Jou T.-S., Juang J.-M.J., Lu J.-Y., Wang I.-J. (2020). Effect of 3-Hydroxy-3-Methyl-Glutaryl-Coenzyme A Reductase Inhibitors on the Meibomian Gland Morphology in Patients with Dyslipidemia. Am. J. Ophthalmol..

[28] Pflugfelder S.C., Tseng S.C., Sanabria O., Kell H., Garcia C.G., Felix C., Reis B.L. (1998). Evaluation of subjective assessments and objective diagnostic tests for diag-nosing tear-film disorders known to cause ocular irritation. Cornea.

[29] Foulks G.N., Bron A.J. (2003). Meibomian Gland Dysfunction: A Clinical Scheme for Description, Diagnosis, Classification, and Grading. Ocul. Surf..

[30] Finis D., Ackermann P., Pischel N., König C., Hayajneh J., Borrelli M., Schrader S., Geerling G. (2015). Evaluation of Meibomian Gland Dysfunction and Local Distribution of Meibomian Gland Atrophy by Non-contact Infrared Meibography. Curr. Eye Res..

[31] Arita R., Itoh K., Inoue K., Amano S. (2008). Noncontact Infrared Meibography to Document Age-Related Changes of the Meibomian Glands in a Normal Population. Ophthalmology.

[32] Wu Y., Jiang H., Zhou X., Zhai Z., Yang P., Zhou S., Gu H., Xu J., Hong J. (2022). Morphological and Functional Changes of Meibomian Glands in Pediatric and Adult Patients with Allergic Conjunctivitis. J. Clin. Med..

[33] Muntz A., Sandford E., Claassen M., Curd L., Jackson A.K., Watters G., Wang M.T.M., Craig J.P. (2021). Randomized trial of topical periocular castor oil treatment for blepharitis. Ocul. Surf..

[34] Vogel R., Crockett R.S., Oden N., Laliberte T.W., Molina L. (2010). Sodium Hyaluronate Ophthalmic Solution Study Group. Demonstration of Efficacy in the Treatment of Dry Eye Disease with 0.18% Sodium Hyaluronate Ophthalmic Solution (Vismed, Rejena). Am. J. Ophthalmol..

[35] Gomez M.-L., Afshari N.A., Gonzalez D.D., Cheng L. (2022). Effect of Thermoelectric Warming Therapy for the Treatment of Meibomian Gland Dysfunction. Am. J. Ophthalmol..

[36] Arici C., Mergen B., Yildiz-Tas A., Bahar-Tokman H., Tokuc E., Ozturk-Bakar Y., Kutlubay Z., Sahin A. (2022). Randomized double-blind trial of wipes containing terpinen-4-ol and hyaluronate versus baby shampoo in seborrheic blepharitis patients. Eye.

[37] Finger P.T., Fam A., Tomar A.S., Garg G., Chin K.J. (2020). Hypochlorous acid antiseptic washout improves patient comfort after intravitreal injection: A patient reported outcomes study. Indian J. Ophthalmol..

[38] Kern J.R., Fahmy A.M. (2019). Dry eye patients report improvement in symptoms with hypochlorous acid use over 30 days. Investig. Ophthalmol. Vis. Sci..

[39] Lee H., Chung B., Kim K.S., Seo K.Y., Choi B.J., Kim T.-I. (2014). Effects of Topical Loteprednol Etabonate on Tear Cytokines and Clinical Outcomes in Moderate and Severe Meibomian Gland Dysfunction: Randomized Clinical Trial. Am. J. Ophthalmol..

[40] Kiamco M.M., Zmuda H.M., Mohamed A., Call D.R., Raval Y.S., Patel R., Beyenal H. (2019). Hypochlorous-Acid-Generating Electrochemical Scaffold for Treatment of Wound Biofilms. Sci. Rep..

[41] Lindsley K., Matsumura S., Hatef E., Akpek E.K. (2012). Interventions for chronic blepharitis. Cochrane Database Syst. Rev..

