# Supplementary material for: Effect of Hypochlorous Acid on Blepharitis through Ultrasonic Atomization: A Randomized Clinical Trial
Source: J Clin Med. 2023 Feb 1;12(3):1164. doi: 10.3390/jcm12031164 (PMC9917691; doi:10.3390/jcm12031164)
Supplement: Supplementary file 1 [file jcm-12-01164-s001.zip › jcm-2169017-supplementary.pdf]

Supplementary Tables for

Effect of Hypochlorous Acid on Blepharitis through Ultrasonic Atomization：A Randomized Clinical Trial

Supplementary Table S1: Changes of clinical outcomes from baseline to 2 weeks after treatment in hypochlorous acid (HOCl) group and control group.

	HOCl group			Control group		
Parameters	Baseline	After 2 weeks treatment	*p* Value	Baseline	After 2 weeks treatment	*p* Value
**Primary outcomes**						
OSDI[a]	35.6 ± 19.3	15.3 ± 9.7	<0.001	31.8 ± 15.3	22.2 ± 11.5	0.001
Lid margin redness[b]	1.5 ± 0.6	0.5 ± 0.5	<0.001	1.7 ± 0.5	1.1 ± 0.3	<0.001
Lid margin abnormality[b]	1.3 ± 0.5	0.5 ± 0.5	<0.001	1.3 ± 0.4	1.1 ± 0.2	0.014
Meibum expressibility[b]	2.3 ± 0.6	1.2 ± 0.4	<0.001	1.8 ± 0.7	1.4 ± 0.6	<0.001
Meibum quality[b]	2.0 ± 0.3	0.7 ± 0.6	<0.001	1.9 ± 0.4	1.3 ± 0.5	<0.001
NIBUT-1st, s[b]	9.2 ± 4.9	11.6 ± 4.7	0.009	7.6 ± 4.9	9.0 ± 4.7	0.009
NIBUT-avg, s[b]	11.6 ± 4.3	13.3 ± 3.4	0.050	10.7 ± 4.2	11.3 ± 4.2	0.040
**Secondary outcomes**						
Conjunctiva redness[b]	1.3 ± 0.6	1.1 ± 0.5	0.002	1.4 ± 0.4	1.3 ± 0.3	0.060
CFS score[b]	0.5 ± 1.0	0.0 ± 0.2	0.010	0.2 ± 0.5	0.2 ± 0.5	0.157
TMH, mm[b]	0.3 ± 0.1	0.2 ± 0.1	0.943	0.2 ± 0.1	0.2 ± 0.1	0.800

Abbreviations: OSDI, ocular surface disease index; NIBUT-1st, the first time of noninvasive breakup time; NIBUT-avg, the mean time of noninvasive breakup time; CFS, corneal fluorescein staining; TMH, tear meniscus height.

[a]*P* values based on paired-t test.

[b]*P* values based on paired-Wilcoxon rank sum tests.

Supplementary Table S2: Changes of clinical outcomes from baseline to 2 weeks after treatment according to baseline meibomian gland loss in the hypochlorous acid (HOCl) group.

Parameters	Baseline mild-moderate MGL n=29			Baseline severe MGL n=6		
	Baseline	After 2 weeks treatment	*p* Value	Baseline	After 2 weeks treatment	*p* Value
**Primary outcomes**						
OSDI[a]	36.6 ± 20.1	15.8 ± 10.2	<0.001	31.0 ± 15.1	12.8 ± 7.0	0.060
Lid margin redness[b]	1.3 ± 0.5	0.4 ± 0.5	<0.001	2.2 ± 0.4	0.8 ± 0.4	0.023
Lid margin abnormality [b]	1.1 ± 0.4	0.5 ± 0.5	<0.001	2.0 ± 0.0	0.8 ± 0.4	0.020
Meibum expressibility [b]	2.1 ± 0.6	1.1 ± 0.4	<0.001	3.0 ± 0.0	1.3 ± 0.5	0.023
Meibum quality [b]	2.0 ± 0.4	0.7 ± 0.6	<0.001	2.0 ± 0.0	0.5 ± 0.6	0.024
NIBUT-1st, s[a]	9.1 ± 5.0	11.9 ± 4.6	0.007	9.4 ± 5.3	10.2 ± 5.6	0.731
NIBUT-avg, s[a]	11.7 ± 4.3	13.3 ± 3.5	0.047	11.0 ± 4.7	13.1 ± 3.2	0.297
**Secondary outcomes**						
Conjunctiva redness[a]	1.2 ± 0.6	1.1 ± 0.5	0.011	1.6 ± 0.6	1.0 ± 0.2	0.096
CFS score[b]	0.5 ± 1.0	0.0 ± 0.2	0.026	0.7 ± 1.2	0.0 ± 0.0	0.180
TMH, mm[a]	0.3 ± 0.2	0.2 ± 0.1	0.297	0.2 ± 0.0	0.2 ± 0.0	0.004

Abbreviations: MGL, meibomian gland loss; OSDI, ocular surface disease index; NIBUT-1st, the first time of noninvasive breakup time; NIBUT-avg, the mean time of noninvasive breakup time; CFS, corneal fluorescein staining; TMH, tear meniscus height

[a]*P* values based on paired-t test.

[b]*P* values based on paired-Wilcoxon rank sum tests.
